# White matter microdissection of the lateral aspect of the brain: 2-dimensional video demonstration

**DOI:** 10.1007/s00701-025-06635-z

**Published:** 2025-08-07

**Authors:** Luca Zanuttini, Victor E. Staartjes, Shao-Ching Chen, Chun-Fu Lin, Sanford P. C. Hsu, Carolina Martins, Hung Tzu Wen, Carlo Serra, Uğur Türe

**Affiliations:** 1 Machine Intelligence in Clinical Neuroscience & Microsurgical Neuroanatomy (MICN) Laboratory, Department of Neurosurgery, Clinical Neuroscience Center, University Hospital Zurich, University of Zurich, Frauenklinikstrasse 10, 8091 Zurich, Switzerland; 2 Department of Biomedical and Neuromotor Sciences (DIBINEM), University of Bologna, Bologna, Italy; 3 Department of Surgery, Division of Neurosurgery, Cheng Hsin General Hospital, Taipei, Taiwan; 4 Division of General Neurosurgery, Neurological Institute, Taipei Veterans General Hospital, Taipei, Taiwan; 5 Neurology and Neurosurgery Unit, Hospital das Clínicas, Federal University of Pernambuco, Recife, Brazil; 6 Division of Neurosurgery, Hospital das Clínicas, University of São Paulo, College of Medicine, São Paulo, Brazil; 7 Department of Neurosurgery, Yeditepe University School of Medicine, Istanbul, Türkiye

**Keywords:** Klingler, White matter, Microneurosurgery, Fiber dissection

## Abstract

**Background:**

The white matter dissection technique owes its modern recognition to Josef Klingler, whose fixation protocol enabled detailed fiber bundle visualization and led to an outstanding and still relevant atlas of white matter anatomy. After a period of decline in the late twentieth century, the technique was reintroduced into neurosurgical training with the aid of the surgical microscope, emphasizing the importance of fiber dissection and its practical applications in clinical neuroanatomy.

This article provides a structured, step-by-step demonstration of white matter microdissection, progressing from superficial anatomy to deeper fiber layers. Its main contribution is the supplementary video, which offers a detailed, structured demonstration of the dissection steps and white matter layers, replicating the format and clarity of hands-on dissection courses*.* Careful editing and post-processing were applied to optimize educational impact.

**Methods:**

The dissection was recorded during the 7th Sulci, Gyri, Ventricles and Fiber Dissection Hands-on Course (Taipei, November 2024). Identifiable participants gave informed consent for publication.

**Results:**

The video illustrates a systematic lateral-to-medial dissection, beginning with sulcal and gyral identification. Grey matter is removed to expose U-fibers, followed by progressive visualization of the superior longitudinal fasciculus, insular cortex, extreme and external capsules, claustrum, basal ganglia, and anterior commissure. Each step is accompanied by commentary and technical tips to guide the dissection and preserve anatomical integrity.

**Conclusion:**

By mastering the topography of white matter, neurosurgeons can enhance their understanding of pathological anatomy, allowing for precise mental rehearsal and strategic planning before surgery. This approach underscores the enduring importance of anatomical studies in improving surgical outcomes and advancing neurosurgical practice.

**Supplementary Information:**

The online version contains supplementary material available at 10.1007/s00701-025-06635-z.

## Introduction

The white matter dissection technique, as it is recognized today, owes its fame to the work of Josef Klingler, a laboratory technician at the Institute of Anatomy in Basel during the first half of the twentieth century [1, 25]. However, the anatomical study of white matter tracts has a long tradition, from Galen’s initial descriptions of the corpus callosum and fornix to the contributions of pioneers such as Vieussens, Malpighi, Willis, Reil, Vicq d’Azyr, and Burdach, who laid the foundations for understanding the gross architecture of the brain’s fiber systems [1, 3, 17, 19, 24, 28, 29]. Klingler’s main contribution was the systematization of a brain fixation protocol based on freezing formalin-fixed specimens prior to dissection, which significantly improved the clarity and precision of white matter fiber dissection [13, 14, 30]. The idea of freezing the brain was strongly inspired by previous authors, particularly by the Viennese school [13]. Despite Klingler's excellent work, which also resulted in a very valuable photographic atlas [14], the interest of the neurosurgical community in the surgical anatomy of white matter waned in the second half of the twentieth century. In the early 2000 s, Türe – inspired by Yaşargil – brought this white matter dissection technique back to the attention of the world literature, highlighting the role of the microscope in a publication that remains a milestone in the field [25]. While Klingler’s method remains the reference standard, more recent studies have introduced refinements to improve fixation homogeneity and anatomical preservation. Notably, intra-carotid formalin perfusion has been proposed to enhance visualization of superficial and deep fiber structures, including vascular and fine associative bundles [16]. Nevertheless, Klingler’s original protocol remains widely adopted, often with slight variations, due to its reproducibility and accessibility [5].

By acquiring knowledge on white matter bundle topography, neurosurgeons can gain a more profound insight into normal and pathological anatomy, thereby supporting mental rehearsal and strategy development prior to surgery. Although several articles and books have described the lateral white matter dissection [10, 11, 14, 15, 25], to our knowledge no detailed video article has been published to date that can guide the reader—or a novice in particular—through the practical steps of this dissection. This video aims to provide a visual guide to the step-by-step procedure for performing a fiber dissection of the lateral aspect of the brain. Furthermore, the video incorporates visual aids to facilitate comprehension of the critical aspects of each step.

## Materials and methods

The proposed white matter microdissection demonstration was recorded during the 7th Sulci, Gyri, Ventricles and Fiber Dissection Hands-on Course, held in November 2024 in Taipei, Taiwan. All identifiable participants provided informed consent for the publication of their images.

The video refers to a single dissection performed by the senior author (C.S.) and was recorded using a Zeiss OPMI Pentero surgical microscope. Video editing and post-processing were carried out using Final Cut Pro (Apple Inc.), which was also used—along with Microsoft PowerPoint—for the creation of schematic overlays.

### Klingler fixation protocol

The Klingler protocol consists of three phases: fixation, freezing and thawing, and dissection. While slight variations are reported in the literature regarding the technical details of each phase, we describe here the protocol currently adopted in the Zurich laboratory, which was also applied to the hemisphere dissected in the presented video.

As originally recommended by Klingler, the brain is removed from the cadaver with the head bent dorsally to obtain a congested specimen, which improves the contrast between grey and white matter [14].

Fixation is performed by immersing the brain, extracted from a fresh autopsy specimen, in a 10% formalin solution for at least two months. To avoid deformation, the brain should be suspended in the fixative, either placed on gauze or hung from the lid of the container by ligating the circle of Willis.

The brain is then refrigerated at a temperature of − 10 to − 15 °C for at least one week. If a longer freezing period is available, we recommend extending it, as it improves the separation of fiber tracts and facilitates the dissection.

The final stage involves thawing the specimen by submerging it in water. The pia mater, arachnoid membrane, and blood vessels are then carefully removed. At this point, the specimen is ready for dissection. Between sessions, the brain is stored in a 5% formalin solution. If dissection is postponed for a month or longer, the specimen should be refrigerated once more for at least 12 h before resuming.

### Brain dissection technique

Based on Klingler’s original recommendations, dissection should be performed using a 2–4 mm wooden spatula to remove the cortex, a watchmaker’s forceps for delicate fiber bundles and subcortical nuclei, and a scalpel to excise large portions of tissue and access deeper regions [13, 14]. He also suggested the use of magnifying spectacles or a perforated head mirror (commonly used by otologists at the time) when unaided vision proved insufficient. In our practice, the entire dissection is conducted under microscopic visualization as proposed by Türe, using either wooden spatulas or suction. DeBakey forceps, watchmaker’s forceps, and a scalpel are valuable tools for specific dissection steps. An irrigator filled with water should be kept at hand to regularly rinse the specimen and maintain its hydration.

## Results

This section outlines the main steps of lateral brain dissection, integrating technical tips derived from the Zurich laboratory’s experience. A detailed demonstration of each step is provided in Supplementary Video 1.

### Step 1 – Detailed study of sulcal and gyral anatomy

The dissection begins with the identification of key anatomical landmarks on the lateral surface of the hemisphere. These include the sylvian fissure, whose horizontal and ascending branches divide the orbital, triangular, and opercular parts of the inferior frontal gyrus, and a posterior branch that extends toward the supramarginal gyrus. The central, precentral, and postcentral sulci delimit the precentral and postcentral gyri. The superior and inferior frontal sulci divide the frontal lobe into the superior, middle, and inferior frontal gyri. The superior and inferior temporal sulci lie between the superior, middle, and inferior temporal gyri. The inferior parietal lobule, formed by the supramarginal and angular gyri, is separated from the superior parietal lobule by the intraparietal sulcus.

### Step 2 – Double-C decortication to expose U-fibers

Decortication is then performed according to the so-called double-C scheme. This involves removing the cortical grey matter to expose the U-fibers—short association fibers connecting adjacent gyri. Dissection is performed along the “inner C” and “outer C”, extending respectively from the inferior frontal sulcus to the superior temporal sulcus, and from the superior frontal sulcus to the inferior temporal sulcus, passing through the central region and inferior parietal lobule. Removal of the cortex should be carried out with a wooden spatula or suction, working from the base toward the crown of each sulcus to minimize damage to the underlying U-fibers. The entire process should be conducted carefully and patiently. For beginners, we suggest starting with the superior temporal sulcus, which is the most accessible. The cortical covering of the opercular gyri should be preserved for the subsequent steps.

### Step 3 – Removal of U-fibers to expose SLF

Beginning at the temporal end, U-fibers from the middle temporal gyrus are gently peeled away to reveal the inferior portion of the superior longitudinal fasciculus (SLF). The SLF is a large, C-shaped association fiber bundle connecting non-adjacent neocortical gyri of the temporal lobe with the occipital, parietal, and frontal lobes, arching around the sylvian fissure. U-fiber removal continues through the parietal and frontal lobes to fully expose the SLF, taking care to identify the correct dissection plane.

Splitting the sylvian fissure allows for complete visualization of the opercula [4]. These cortical regions, whose name derives from the Latin"operculum"(cover), form the roof of the insula, which has a pyramidal shape with an inferolateral apex and is bounded by the periinsular sulci. The opercula can be divided in three parts by the posterior and horizontal rami of the sylvian fissure. The horizontal ramus of the sylvian fissure separates the frontoorbital operculum from the frontoparietal operculum, while the posterior ramus separates the latter from the temporal operculum.

The frontoorbital operculum is separated from the insula by the anterior periinsular sulcus and is composed laterally of the posterior and lateral orbital gyri and the pars orbitalis of the inferior frontal gyrus; medially, it includes the anterior and posterior suborbital gyri. The frontoparietal operculum, separated from the insula by the superior periinsular sulcus, comprises the pars triangularis and opercularis of the inferior frontal gyrus, the lower parts of the pre- and postcentral gyri, and the superior supramarginal gyrus laterally; medially, it includes the subtriangular, subopercular, subprecentral, and subcentral gyri, as well as the anterior, middle, and posterior transverse parietal gyri (the last three not visible in this dissection). The temporal operculum, separated by the inferior periinsular sulcus, includes the superior temporal gyrus and the inferior branch of the supramarginal gyrus laterally, and medially comprises an anterior flat portion, named planum polare, followed by the transverse temporal gyri and a posterior flat portion, named planum temporale.

### Step 4 – Removal of the opercula to expose the insula

Using a scalpel, the opercula are carefully removed in a piecemeal fashion down to the insular limiting sulci. The insular cortex, now fully exposed, is divided into an anterior (larger) and posterior (smaller) portion by the central insular sulcus, which parallels the central sulcus on the lateral surface. The anterior part contains the anterior, middle, and posterior short gyri, separated by the short insular and precentral insular sulci. These gyri converge at the insular apex. Anterior to them are the accessory insular gyrus, beneath the frontoorbital operculum, and the transverse insular gyrus, extending from the insular apex toward the posteromedial orbital lobule. The limen insulae marks the transition between the insula and the anterior perforated substance. Posteriorly lie the anterior and posterior long gyri, separated by the postcentral insular sulcus [26].

### Step 5—Insular decortication to expose the extreme capsule

The thin insular cortex is then removed using the same technique exposed in the first step to expose the extreme capsule, a thin layer composed primarily of U-fibers arching between the insular and opercular cortices.

### Step 6 – Removal of the extreme capsule to expose the claustrum and the external capsule

By gently removing the fibers of the extreme capsule, the claustrum and the external capsule are revealed. The claustrum appears as a thin layer of grey matter situated between the extreme and external capsules. The external capsule can be subdivided into a dorsal part, which is composed primarily of vertically oriented claustrocortical fibres, and a ventral part, which is composed of the fronto-occipital fasciculus (FOF) extending from the frontal to the occipital region, and the uncinate fasciculus (UF) connecting frontoorbital and temporopolar cortices. These two associative bundles course from the temporal to the frontal lobes, forming a narrow isthmus beneath the limen insulae, where they cannot be easily distinguished from each other.

For dissection purposes, the fibers of the extreme capsule can be differentiated from those of the external capsule based on their trajectory: the former run lateral to the SLF toward the opercula, while the latter course medially. To better expose the underlying structures, a scalpel is used to make a sharp incision at the arching portion of the SLF, after which the lower part of the fasciculus is carefully peeled away, revealing the posterior extent of the FOF.

### Step 7 – Removal of the claustrum and external capsule to expose the putamen

The thin layer composed by the claustrum and external capsule is removed to expose the putamen and, superiorly, the fibers of the internal capsule. The putamen and globus pallidus together constitute the lentiform nucleus, as defined morphologically.

### Step 8 – Removal of the putamen to expose the globus pallidus and the internal capsule

The putamen is then removed, preferably using suction due to its texture, to expose the firmer and paler globus pallidus. These two nuclei differ in texture and color, with the term “pallidus” derived from Latin for “pale”.

### Step 9 – Exposure of the anterior commissure

In the final step, the anterior commissure is exposed. The floor of the cavity left by putamen removal is perforated by vertically oriented lenticulostriate arteries, which traverse the anterior perforated substance. Just posterior and superior to these perforators, the fibers of the anterior commissure can be observed passing beneath the globus pallidus and spreading laterally into the anterior temporal region. These fibers run through a tubular depression below the globus pallidus known as the Canal of Gratiolet. To visualize this structure, the inferior globus pallidus is carefully removed. The thick bundle of commissural fibers forming the medial anterior commissure is then clearly visible, linking temporal lobe structures across hemispheres and passing beneath both the FOF and UF.

## Discussion

The Klingler technique, developed in the 1930 s, remains the most widely adopted protocol for brain preparation in order to perform white matter dissection due to its reproducibility and results in term of fiber separation. The rationale for its success lies in the freezing phase, which causes water and formalin to transition from liquid to solid, thereby expanding in volume and promoting the separation of fiber bundles. Over the years, as suggested by a recent review on the topic, several refinements of the three phases of the protocol have been proposed [5]. Variations have been reported regarding the interval between death and brain extraction (ranging from 8 to 72 h), the concentration of formalin used for fixation (4% to 15%), as well as freezing temperatures (–5 °C to –80 °C) and durations. Notably, longer freezing times generally improve the cleavage of white matter fibers. Türe and colleagues reintroduced the technique into neurosurgical practice with the addition of microsurgical tools, such as the operating microscope, which allows for greater precision and three-dimensional appreciation of tracts [25]. Other authors have compared different fixation strategies, underscoring the importance of intra-carotid formalin perfusion to improve deep tissue fixation [16]. Notably, the deeper structures of the brain are often inadequately preserved by immersion alone. Klingler himself recommended using a 5% formalin solution rather than 10% to facilitate deeper penetration, albeit at the cost of prolonged fixation times, which may compromise specimen quality due to progressive tissue degradation. In this context, intra-arterial perfusion offers a valuable alternative, although it requires a specifically equipped facility.

These technical adaptations continue to shape the application of Klingler’s method in contemporary anatomical and surgical training. Notably, there is currently no objective evidence clearly identifying a superior variant of the"Klingler protocol,"and most adjustments remain based on cumulative experience within individual laboratories [5]. Comparative studies—evaluating, for instance, different freezing temperatures and durations—could help define optimal parameters for fiber tract separation and visualization. As in other institutions, we rely on a protocol that has proven most effective in our setting. Of note, we have recently observed that, when dissections are carried out over several days, re-freezing the specimen overnight between sessions appears to improve fiber bundle cleavage. This exemplifies how empirical observations in daily practice may progressively inform protocol refinements.

The fiber dissection of the lateral brain surface provides an effective way to explore the three-dimensional organization of white matter tracts. This anatomical knowledge is essential for neurosurgeons, enhancing their understanding of normal neuroanatomy and how fiber pathways may be displaced or infiltrated by pathological processes [30]. While several studies and atlases have described these dissections [4, 6, 7, 10, 11, 15, 18, 21, 23, 25–27], the technical execution is often limited to hands-on courses or anatomical labs.

Our video aims to address this gap with a step-by-step demonstration enriched by visual markers and commentary, allowing clear visualization of dissection planes and progressive exposure of deeper structures. This immersive approach may prove particularly beneficial for trainees and clinicians unfamiliar with fiber dissection techniques.

Although recent advances in virtual and augmented reality are reshaping neuroanatomical education [2, 8, 9, 20], their value remains complementary. These technologies, while promising, cannot yet precisely replicate the tactile feedback, spatial depth, and three-dimensional physical rendering that are intrinsic to hands-on cadaveric dissection. This limitation is particularly evident in white matter anatomy, where the perception of fiber orientation, consistency, and resistance is crucial. Direct dissection, presented here through a video demonstration, continues to offer an irreplaceable educational experience, fostering both technical proficiency and a deeper understanding of neuroanatomical topography.

Furthermore, as neurosurgical approaches to deep-seated lesions continue to evolve [12, 22, 31], the ability to mentally reconstruct white matter anatomy becomes increasingly relevant to improving surgical planning, reducing functional risks, and optimizing patient outcomes.

This work, however, presents some limitations. It reflects the experience and protocol of a single anatomical laboratory, and dissection outcomes may vary depending on specimen preparation, anatomical variability, and operator expertise. Furthermore, although the video was edited to enhance the comprehension of three-dimensional white matter anatomy, it cannot fully replace hands-on dissection. Rather, it should be viewed as a complementary tool to support the acquisition of initial know-how and preparation for practical training.

## Conclusion

In summary, this original article, with its core supplementary video, presents a comprehensive, stepwise demonstration of lateral white matter dissection using the Klingler technique. By offering a dynamic visual guide to each anatomical layer, we aim to facilitate both basic neuroanatomical understanding and advanced surgical planning. We believe that accessible, high-quality educational resources such as this video can play a crucial role in training the next generation of neurosurgeons and supporting continuous learning among experienced practitioners.

## Supplementary Information

Below is the link to the electronic supplementary material.

Supplementary file1 (MP4 970882 KB)

## Data Availability

No datasets were generated or analysed during the current study.
